# Are Narrative CVs contributing towards shifting research culture? Workshop Report from the 2023 Recognition and Rewards Festival

**DOI:** 10.12688/f1000research.146108.1

**Published:** 2024-04-23

**Authors:** Noémie Aubert Bonn, James Peter Morris, Sean Sapcariu, Karen Stroobants

**Affiliations:** 1 Department of Healthcare and Ethics, Universiteit Hasselt, Hasselt, Flanders, 3500, Belgium; 2 Science Europe, Brussels, Brussels, 1040, Belgium; 3 Fonds National de la Recherche, Luxembourg City, Luxembourg District, 4365, Luxembourg; 4 CultureBase Consulting, Cambridge, UK

**Keywords:** Research Assessment; Research Culture; Qualitative Assessment; Narrative CV; Diversity; Research Careers

## Abstract

**Background:**

Over the past decade, calls for research assessment reform have grown, led by initiatives such as the Declaration on Research Assessment (DORA) and the Leiden Manifesto, and, more recently, the Coalition for Advancing Research Assessment (CoARA). A key element being discussed as part of research assessment reform is a shift towards more qualitative assessments, focussed on the content of research and the broad skills and competencies of researchers, and the array of contributions they make to knowledge creation and innovation. Narrative CV formats have emerged as a good practice example for enabling qualitative assessments of research projects and researchers, and are becoming more widely piloted and implemented.

**Methods:**

As part of the 2023 Dutch Recognition and Rewards Festival, the authors hosted a workshop to gather perspectives on Narrative CVs, including whether and how they may contribute to shifts in research culture that are needed to support research assessment reform.

**Results:**

Participants, representing research organisations and the research community, discussed both beneficial and critical aspects of narrative-style CV implementations from their experiences. The effects observed since narrative CVs have been implemented were discussed, with perspectives provided on career prospects and the empowerment of the research community to direct change. Finally, the discussion turned to expectations for the future, with workshop participants calling for focus on the roles that narrative-style CVs can play in improving research careers, recognition of collaborative work, and equality, diversity, and inclusion. A short informal survey exploring levels of implementation of narrative CVs across different research organisations was run prior to the workshop, the results of which are also presented as part of this report.

**Discussion:**

The authors intend to expand this discussion to other scientific and policy conferences, and this report serves as a basis for a wider and deeper dialogue in the community.

## Introduction

Over the past decade, calls for research assessment reform have gained great momentum. Initiatives such as the Declaration on Research Assessment ^1^ (DORA) and the Leiden Manifesto ^2^ raised considerable awareness about the need to rethink research assessment and were rapidly followed by a multitude of reports, statements, and recommendations from national and international organisations. ^3^^–^^12^ More recently, the interest in changing research assessment even acquired a global scope, with organisations such as the G7, ^13^ UNESCO, ^14^ and the Global Research Council, ^15^ addressing the topic formally. The Coalition for Advancing Research Assessment ^16^ (CoARA) was created against this background, aiming to provide support and coordination to the global reform of research assessment. CoARA already counts the support of nearly 600 research organisations (as of November 2023), highlighting a true momentum for change.

A key element being discussed as part of research assessment reform activities is a shift towards more qualitative assessments, focussed on the content of research and the broad skills and competencies of researchers, and the array of contributions they make to knowledge creation and innovation. Narrative CV formats have emerged as a good practice example for enabling qualitative assessments of research projects and researchers, and are becoming more widely piloted and implemented.

## Methods

### Ethics

Given the scope of this report and associated workshop, the workshop was conducted as an exploration from interested organisations without seeking ethical approval. Participants agreed to take part after being made aware that a summary of the workshop would be published from the discussions.

### Methodology

On April 13 ^th^, 2023, we held a workshop with the title *“Are narrative CVs proving effective in achieving desired outcomes of recognition and reward initiatives*?” at the Recognition and Rewards Festival in Utrecht, NL in order to gather perspectives on the role that narrative CVs play in shifting research cultures and in enabling the research assessment reform. This report summarises the views and opinions expressed by the 14 participants that joined the workshop, and serves as a resource for others in further reflections and development of narrative-style CV templates. Participants included research funders and three researchers, most of them active in the Dutch research landscape. The feedback in this report should help guide organisations and institutions considering or implementing narrative-style CVs when thinking about the potential impact on research culture that could occur due to these changes. In addition, we hope that our experience can be a template for other workshops around this topic, and that this report serves as a catalyst for further dialogue and proactive communication around narrative-style CVs and their impact on research culture.

## Results

### Views on narrative CVs/challenges with their use

The workshop participants raised many beneficial and critical aspects of the narrative-style CV, all of which need to be considered by funders and institutions who are implementing these new formats. However, the general agreement was that effective and efficient assessment reform will take time and requires systemic change, due to the many different parties involved in changing assessment, above and beyond only changing a CV format.

In general, narratives were seen to have always been a part of an evaluation, whether or not they were in the CV. In this sense, a narrative was seen as required for an evaluation – the narrative-style CV helps in increasing the understanding of a candidate and legitimising the contributions that were previously not well-recognised since it requires the researcher to tell their own story about their career and achievements. At the same time, this increased flexibility can be confusing, so balancing structure and flexibility in the formats is needed. This can be a different way of evaluating compared to the current process, leading to difficulties for reviewers in terms of comparing applicants and assessing the content.

Participants felt that new CV formats can lead to increased use of buzzwords and a loss of authenticity, as they might feel obligated to fill in all of the fields of a template, to “tick all the boxes” and thus get an edge in the evaluation. Certain participants stated that a need to appear competent everywhere might create a loss of authenticity for researchers. While it is important for institutions using a narrative-style CV to emphasise that what is requested is not an increased number and range of accomplishments, participants also argued that a lack of authenticity is generally identifiable in an evaluation (especially an interview), and as such would not prove to be a problem in the long term adoption of new CV formats.

One of the concerns raised was the perspective that there is not much uptake in narrative-style CV use internationally, and the widespread use of quantitative data and metrics is still present in systems around the world. Given our informal pre-workshop survey results (see Box 1), this perspective might reflect a lack of awareness of the international landscape and use of narrative-style CVs. Regarding the engagement with the format, applicants were told specifically not to add certain (less traditional) contributions because they could be perceived as a “lack of focus”. This shows a clear resistance from a part of the academic community for the narrative-style CV, and a need for more dialogue around these changes.

Box 1. **Informal pre-workshop survey.**

**Methods**

A short 12-question survey was devised by the report authors and distributed to representatives from research organisations via the following forums: the DORA Funders Group; the Global Research Council Responsible Research Assessment Working Group; and the Science Europe Working Group on Research Culture.

The survey was launched between March and April 2023 and received 24 full responses from representatives of 23 research organisations, covering 18 countries across 4 continents and one European-wide organisation. The aggregated data were presented during the workshop at the Recognition and Rewards Festival in Utrecht, NL on 13 April 2023 solely to provoke discussion and are not used for other purposes. The results are presented in this report in the same manner. The survey data were aggregated to avoid re-identification. Raw results data cannot be shared as individual responses would easily allow for identification of the respondent in several cases. The questions asked in the survey, a mix of multiple choice and open text, are provided in the codebook that is available alongside the data published at https://doi.org/10.6084/m9.figshare.25146155.v1.

**Short summary of survey results**

Across the 24 respondents to the survey, and in response to both open-ended and multiple choice questions (see annex 1 for the full list of questions), just over half of the responding organisations (54 %) had already implemented a narrative CV format as part of their assessment processes. These organisations were spread across different regions of the world, including Latin America and the Caribbean, Australia and New Zealand, Northern America, and Northern, Western, and Southern Europe. Among these organisations, there was a split between those that implemented the same narrative CV format across all programmes (42 %) and those that implemented the format only for specific programmes (58 %). The most commonly reported approach to implementing a narrative CV was to develop a template ‘in house’, from scratch (54 %). Less commonly, organisations reported building upon an existing template, adapting it to their needs (30 %), and just 2 organisations (15%) reported using existing templates with little adaptation. The Royal Society’s Narrative CV template, the “Résumé for Researchers” was cited by a number of respondents as the guidance template used.

When asked what the main reasons or goals for introducing a narrative CV format were, respondents referenced: broadening the recognition of research activities, contributions, and skills; supporting equality, diversity, and inclusion; building compliance with community-driven initiatives such as DORA; and simply as an attempt to improve the evaluation and selection of researchers and research projects.

**Figure 1.**
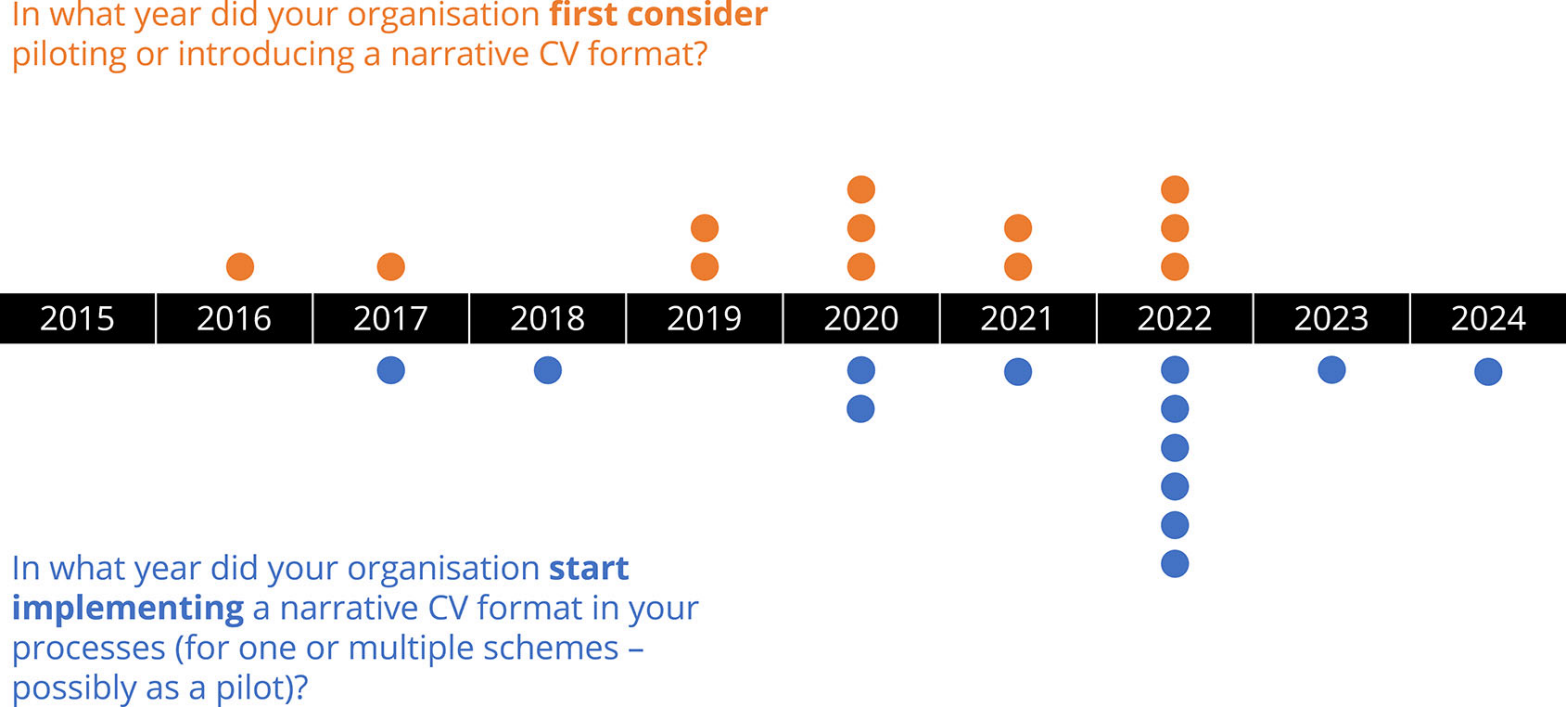
Research organisations that implement a narrative CV format were asked when they first considered piloting or introducing a narrative CV format, and when they started implementing it as part of their assessment processes.

A wide spread of responses were obtained in response to the question about the years when organisations first considered piloting or introducing a narrative CV format, beginning from 2015, and most commonly around 2020. Subsequent implementation of a narrative CV as part of assessment processes was observed to follow quickly after first consideration, often only taking one or two years (see Figure 1). Some common challenges to implementing narrative CVs were reported by responding organisations, including: 1) resistance to change, 2) difficulty in developing appropriate training and guidance, and 3) clear and effective communication on the reasons for change.

Responding organisations that had not yet implemented narrative CVs (n = 11) within any part of their assessment processes shared diverse reasons why they had not. A number of respondents were considering implementing them in the future. Others reported that it was not a priority for the moment. Some respondents reported a current lack of data to support the effectiveness of such a change as a barrier whilst other respondents cited administrative challenges.

Note: We omitted one respondent in the first question since they mentioned that they always used narrative CVs for hiring.

It was mentioned that narrative-style CVs have the possibility of helping with increasing the diversity of who is funded, specifically with females and minority groups. The new format allows people to describe a wider diversity of careers and outputs, facilitating inclusion for people with different backgrounds. However, at the same time, the quality of narratives are dependent on writing ability, which could introduce new biases and requirements for support and training – something that not all people and institutions have the resources to implement.

The introduction of the narrative-style CV is seen as an iterative process with regards to the structure and content requested, as well as the openness of the template. As the needs and focus of the different organisations and the goals of assessments are different, participants felt that a tailored approach is needed for the various narrative-style CV templates. At the same time, participants stressed that funders and institutions should not judge academics beyond what is required for the remit of the assessment, and should take into account potential biases arising from the new format.

### Changes as a result of introducing narrative CVs

Introduction of narrative-style CVs and other initiatives to diversify and broaden the ability of researchers to demonstrate their achievements have led to concrete changes in the perspectives and careers of people in the community, both with positive and negative effects. Participants discussed the reflection required in filling out a narrative CV being a positive experience, and the need for reflection to be present earlier in academic trajectories. In addition, one participant described a narrative-style CV playing a role in the promotion of people that would have been unlikely to have this opportunity otherwise. Another participant highlighted the role that narrative-style CVs could play in empowering researchers to contribute to research culture shifts by allowing them to describe competencies, activities, or actions that they deem important to the research culture they want to work within.

One issue raised was around the communication of the shift to a narrative-style CV. The request for a broad range of output and achievements in evaluation was viewed by researchers as a requirement to fulfil the entire breadth of outputs and achievements that can be accounted for. This highlights a need to ensure that researchers are aware of the requirements around narrative-style CVs, through training of applicants and assessors, fostering dialogue around these new forms, and other avenues of proactive communication. Guidance documents are a useful starting point, but are best complemented by active reinforcement, as they are passive tools and are not always read as carefully. As mentioned above, not everyone has an equal level of writing ability, and institutions should offer support where possible to ensure that there is no disadvantage in writing narrative-style CVs.

Participants highlighted that data collection around the myths and assumptions of narrative-style CVs will also help to ensure clarity of these new evaluation tools. They also suggested that these new formats will cause power dynamics to shift, yet it is not quite clear what this will mean in terms of the evolution of research ecosystems. These shifts should be monitored closely to ensure that the changes fit the research culture that is desired.

### Expectations for the future

As this shift towards narrative-style CV uptake in assessment processes continues, participants gave their opinions on how this change should be handled, with specific goals and needs they would like to see. These expectations address specifically the culture change that is required to ensure a diverse and inclusive research ecosystem where people are evaluated on a broader range of research outputs, contributions, and achievements.

In general, participants expressed a need to not be naive in implementing changes, and to try and keep everyone on board through the shift in the research ecosystem. The importance of all roles required in research (e.g. HR staff, technicians, etc.) should be highlighted, and all of these voices should be included in system changes. To this end, a broader diversity of career trajectories should be considered when shifting assessment (and the skills that come along with these different career paths), including careers with inter-sectoral mobility as well as more “classical” career paths.

A need for more diversity and inclusion was requested, specifically in the creation of research systems that are beneficial to younger individuals in research, as well as women and minorities. This can be done through creating more diverse evaluation panels and hiring/promotion assessment committees, as well as by engaging a more diverse group of voices in the dialogue around system adaptation and change. Epistemic diversity should also be taken into account, in order to break the current dominance of “excellence” that reinforces hierarchies between disciplines.

Shifting research culture, including spreading the use of the narrative-style CV, requires bottom-up involvement and peer exchange from individuals in research. Collaboration is an important value in research culture, and participants expressed a wish to share their experiences with others. At the same time, adequate support from institutions and funders for these new formats should be put in place. Funders and institutions need to make sure that changes do not impact the performance of research – we need to balance the time and resource commitment required in assessment with the desire for more creative ideas and faster innovation.

## Discussion

It is vital that discussions targeting changes to research assessment practices involve all members of the research community. This report describes results from a workshop that aimed at being the start of an ongoing and self-reflecting discussion on the topic of narrative CVs as contributors to positive shifts in research culture. The workshop, hosted as part of the Dutch Recognition and Rewards Festival (2023), was attended by a group of engaged and reform-supportive stakeholders from research organisations, research funders, and the research community. It provided a first snapshot of perspectives that will be built on through further future events.

Reflections by researchers who participated in the workshop emphasised the role that narrative-style CVs can play in broadening the types of activities, contributions, outputs, and achievements that are recognised and how this can translate into a more diverse selection of people and ideas as part of our understanding of high-quality research. They added that the process of completing a narrative CV may in itself provide insights and inspiration on the diversity of activities that are perceived as important in research careers, and that introducing narrative CVs may play a role in shaping new cultures of success and achievements among researchers. Yet the discussion also provided insights on elements that need to be considered in order to ensure that narrative CVs achieve their desired purpose. Key learnings from the workshop discussions included an understanding of existing inequalities in the support (through training and guidance, for instance) provided by research institutions towards narrative CV development, and a lack of clear descriptions of the breadth of activities and outputs that can be included (and how they are assessed). Such inequalities should be scrutinised and minimised so as to avoid introducing additional bias into assessment processes. Workshop participants were not well-aware of the growing interest and uptake of narrative-style CVs and qualitative assessment across research organisations in Europe and beyond: a trend being pushed by community-led initiatives such as DORA and CoARA. Research organisations may consider how they effectively communicate and engage with research community members to shed light on the work and changes taking place to support the evolution of research cultures and promote quality research. As these changes to policies and practices become further embedded in our research systems, it is vital that they are regularly discussed, reviewed, and analysed to identify and mitigate unintended and unwanted consequences that may arise for any constituency within our research communities. Overall, the workshop highlighted the value and importance of engaging community members in research policy and practice discussions, and the need for continued dialogue.

It is important to note that this change process is long and complex, and narrative-style CVs are just one part. We need to prepare for what the future will bring in research assessment, and make sure that the community actively mitigates the issues that might arise, including the impact of new technologies on assessment and the creation of new inequalities based on different players changing the system at different speeds. A higher-level and global view will be crucial to ensure that the ecosystem is steered towards a diverse and inclusive research culture, which will require all of the participants in the system to work together towards this goal.

To this end, we encourage more conversations and debate around this topic, as only through dialogue will we be able to create a research culture that recognises and embraces everyone who contributes to the research ecosystem. Workshops like this help to make people more aware of the shifting ecosystem as well as to ensure that a diverse range of voices contribute to these shifts. We propose holding these workshops at a variety of scientific and policy conferences in many domains, so that as many people as possible can contribute to the discussion. If you are organising, participating in, or aware of an event where a structured discussion on Narrative CVs and research culture would be welcome, do not hesitate to contact the authors to explore how a workshop like the one described above could be incorporated or proposed.

## Data Availability

Figshare: Data on the implementation of Narrative CV captured ahead of the 2023 Recognition and Rewards Festival, https://doi.org/10.6084/m9.figshare.25146155.v1. ^17^ This project contains the following underlying data:
• Codebook.pdf • Codebook.xlsx • Data.csv Data are available under the terms of the Creative Commons Attribution 4.0 International license (CC-BY 4.0).
