# Supplementary material for: Are Narrative CVs contributing towards shifting research culture? Workshop Report from the 2023 Recognition and Rewards Festival
Source: F1000Res. 2025 Jun 19;13:332. Originally published 2024 Apr 23. [Version 2] doi: 10.12688/f1000research.146108.2 (PMC12417976; doi:10.12688/f1000research.146108.2)
Supplement: Peer review history

# Reviewer report: Reviewer response for version 2

**DOI:** 10.5256/f1000research.183663.r405115

**Published:** 2025-09-10

**Authors:** Ruth Schmidt

**Affiliations:** 1 Illinois Institute of Technology, Chicago, USA

This brief report articulates views on the state of narrative CVs that were collected from a workshop and survey; while the authors recognize that the limited number and fairly narrow composition of these views might over-index on some items while underplaying others, they posit (and I agree) that this efforts like these are valuable in identifying areas for further inquiry to ensure that narrative CVs can be more effectively employed in assessing research and researchers.

 The points raised are well-taken and provide a good landscape of many issues facing uptake, use, and potential issues with the narrative CV format, which holds promise as a way to reduce reliance on traditional quantifiable metrics, but also introduces the potential for new challenges and barriers to adoption.

 Within the report, the wide variety of insights and observations raised by the authors are all relevant and interesting but feel a bit unstructured in some places and redundant in others. This makes it harder than necessary to walk away with a cohesive view on the themes raised. My main recommendation, therefore, is primarily oriented toward clarifying dominant themes within (or across) the major sections such that a reader can more easily digest the useful content.

 For example, the *Views on narrative CVs/challenges with their use *section represents many findings that are each individually interesting, but which might be largely clustered into four major themes: narrative CV’s potential to engage an increased diversity of people and backgrounds; the need to balance flexibility and structure; the potential for bias on the part of both researchers and assessors; and headwinds that might discourage uptake. Stating these themes before introducing the more detailed findings will help a reader first grasp higher-level principles, which can then be expounded upon with the more concrete findings.

 Similarly, *Changes as a result of introducing narrative CVs* seems to center on three main ideas: the potential for unexpected positives; the need for support/training for both researchers and assessors; and the need to collect/evaluate implications and value that arise from use. *Expectations for the future *centers primarily on seeing NCVs as an iterative, collective effort — top-down and bottom up, with diverse views and trajectories — if it is to yield collective benefits. Finally, the *Discussion* summarizes five main prongs, some of which pull from sections above: possible (unexpected) benefits of CVs; the need for thoughtful support and communication to avoid amplifying known inequities or creating new ones; areas for further research and analysis; the need for open, diverse,  and continual communication; and recognizing that narrative CVs require a systems approach.

 In each of these sections, the addition of even a single sentence stating these kinds of overriding themes (with possible minor reorganization of a point here or there) would increase the chances that a reader comes away with a more coherent sense of the major issues and possibilities, and — perhaps more importantly — can help make those findings ‘stickier’ and more memorable such that they take these very useful insights forward into other conversations and activities in the responsible research assessment space.

Is the work clearly and accurately presented and does it cite the current literature?

Yes

If applicable, is the statistical analysis and its interpretation appropriate?

Not applicable

Are all the source data underlying the results available to ensure full reproducibility?

Yes

Is the study design appropriate and is the work technically sound?

Yes

Are the conclusions drawn adequately supported by the results?

Yes

Are sufficient details of methods and analysis provided to allow replication by others?

Yes

Reviewer Expertise:

Responsible research assessment reform; qualitative research; behavioral systems system

I confirm that I have read this submission and believe that I have an appropriate level of expertise to confirm that it is of an acceptable scientific standard.

# Reviewer report: Reviewer response for version 2

**DOI:** 10.5256/f1000research.183663.r405118

**Published:** 2025-09-08

**Authors:** Marianne Gauffriau

**Affiliations:** 1 IT University of Copenhagen, Copenhagen, Denmark

The manuscript presents a report from a workshop in 2023 on narrative CVs hosted by the authors. Narrative CVs are presented in the context of the research assessment reform. In the workshop, 14 participants (research funders and researchers) discussed the use and future of narrative CVs. To initiate the discussion, the authors had, prior to the workshop, conducted a survey with 24 respondents from research institutions. The survey is also reported in the manuscript.

 Findings from the workshop and the survey give nuanced illustrations of the perception of narrative CVs and their use. The manuscript gives examples of how pros and cons of narrative CVs were discussed in 2023. This documentation is an important contribution for future work on narrative CVs. Topics discussed by the workshop participants can serve as inspiration for new empirical studies, and thus improve our understanding of how narrative CVs work.

 I have two suggestions for clarifications that may strengthen the manuscript:

 1) The Introduction gives a brief and accurate introduction to the research assessment reform supported by relevant literature references. The second part of the Introduction is about narrative CVs with a good description of the CVs. However, I miss a short state-of-the-art with relevant literature references up until 2023 on narrative CVs. This will be important for future studies. Furthermore, a state-of-the-art can elaborate on the statements in the Introduction "Narrative CVs are started to be implemented quite recently, a lot remains to be known about their impact and potential" and in the Discussion "… the growing interest and uptake of narrative-style CVs and qualitative assessment across research organizations in Europe and beyond: a trend being pushed by community-led initiatives such as DORA and CoARA". The latter is supported by the informal pre-workshop survey. Do other studies arrive at similar results?

 2) The survey design ans results are well documented with access to codebook and data. However, information about the workshop design is missing. For example, access to the workshop program and a brief discussion of methodological choices would be beneficial and indeed support the authors’ suggestion "that our experience can be a template for other workshops around this topic".

 Narrative CVs and their potential in the implementation of the research assessment reform is an important and timely topic relevant to many stakeholders in academia and beyond.

Is the work clearly and accurately presented and does it cite the current literature?

Partly

If applicable, is the statistical analysis and its interpretation appropriate?

Yes

Are all the source data underlying the results available to ensure full reproducibility?

Yes

Is the study design appropriate and is the work technically sound?

Yes

Are the conclusions drawn adequately supported by the results?

Yes

Are sufficient details of methods and analysis provided to allow replication by others?

Partly

Reviewer Expertise:

research evaluation, responsible research assessment, bibliometrics

I confirm that I have read this submission and believe that I have an appropriate level of expertise to confirm that it is of an acceptable scientific standard, however I have significant reservations, as outlined above.

# Reviewer report: Reviewer response for version 2

**DOI:** 10.5256/f1000research.183663.r393273

**Published:** 2025-07-09

**Authors:** Simon Kolstoe

**Affiliations:** 1 School of Health & Care Professions, University of Portsmouth, Portsmouth, England, UK

I'm happy that they have made some thoughtful additions to the paper and engaged with the peer reviews. I have no further comments to make.

Is the work clearly and accurately presented and does it cite the current literature?

Yes

If applicable, is the statistical analysis and its interpretation appropriate?

I cannot comment. A qualified statistician is required.

Are all the source data underlying the results available to ensure full reproducibility?

Partly

Is the study design appropriate and is the work technically sound?

Yes

Are the conclusions drawn adequately supported by the results?

Partly

Are sufficient details of methods and analysis provided to allow replication by others?

Yes

Reviewer Expertise:

Originally a Biochemist, now Associate Professor of Bioethics with a specific interest in research integrity, ethics and governance.

I confirm that I have read this submission and believe that I have an appropriate level of expertise to confirm that it is of an acceptable scientific standard.

# Reviewer report: Reviewer response for version 1

**DOI:** 10.5256/f1000research.160148.r336924

**Published:** 2024-11-25

**Authors:** Simon Kolstoe

**Affiliations:** 1 School of Health & Care Professions, University of Portsmouth, Portsmouth, England, UK

***Narrative CVs - where next on the journey?***

 Who your audience is, and what you are trying to communicate to that audience, is the most important consideration for authors of any type of document. When writing an academic CV the audience is often an employment or grants panel who will be looking for indications that the author will meet whatever criteria they have established for the role/funding. The problem is that the criteria is not always explicit, and can often reflect unrecognised prejudice, making it quite a challenge to write a CV that "plays the game" well, especially for people with diverse or non-traditional backgrounds.

 To address this a number of "key performance indicator" (KPI) style metrics have been proposed for academics/researchers, and although they are widely used, they are open to significant manipulation. They are also quite biased towards indicators that current senior researchers value in their own CVs, rather than indicators that might lead to better research overall.

 This weakness of the KPI approach has been increasingly recognised over recent years thanks to an encouraging international effort to consider issues relating to research integrity and culture in order to highlight the broader virtues and responsibilities of a good researcher. The development of narrative CVs is one attempt to recognise this by using, as the authors of this manuscript state, a qualitative rather than quantitative approach.

 However, as with many issues in life, a pendulum swing from one extreme (quantitative indicators) to the other (qualitative indicators) is not the answer. The solution to fair assessment most likely sits somewhere in the middle, with this middle being identified through further discussion and reflection on what we do now, what works or doesn't, and how we might change systems and processes. This is precisely the conversation that this manuscript addresses.

 Viewing this work as a reflection on narrative CVs is helpful, because the sample size is small. Indeed with only 24 responses to the survey I would like to see the authors report number of responses in their summary of survey results rather than percentages to better reflect the very small sample. Likewise the 14 participants in the workshop cannot in anyway be considered representative of the research community, and this should be acknowledge explictly. Thus said a very small sample does not prevent interesting observations being made.

 One element of the results/discussion I did find interesting was the fear that narrative CVs may favour more talented writers, potentially coming from English speaking education systems. This is a fair comment and concern, but I would also like to see the author address the recent challenge raised by the use of AI for both proof reading and also creating content. Surely this must be an increasing threat to the idea of a narrative CV, and even if it wasn't mentioned by participants in the workshop, the author could raise the fact that this wasn't discussed and perhaps should have been.

 I was also struck by the comment that *"Epistemic diversity should also be taken into account, in order to break the current dominance of “excellence” that reinforces hierarchies between disciplines"*. I think this relates to a perceived prejudice towards medical and physical sciences that many from a social science background are often quite concerned about. However, as many CVs often function to obtain positions/grants etc. within subject areas, and thus are judged by academics of similar backgrounds, I would be interested to hear some real examples of where Epistemic diversity is important.

 The Discussion starts with the sentence: " *It is vital that discussions targeting changes to research assessment practices involve all members of the research community.*" I'm not certain this sentence is needed, or indeed justified without further explanation - who is the research community, does it involve research participants, government ministers etc. and should they all be involved in this conversation? Perhaps this sentence could be deleted or alternatively justified further.

 The second paragraph of the discussion also repeats many of the results so could either be deleted or reframed to make a specific, explicit, point.

 But overall this manuscript represents an interesting reflection on the topic, and should be considered as a valuable contribution to the ongoing discussion.

Is the work clearly and accurately presented and does it cite the current literature?

Yes

If applicable, is the statistical analysis and its interpretation appropriate?

I cannot comment. A qualified statistician is required.

Are all the source data underlying the results available to ensure full reproducibility?

Partly

Is the study design appropriate and is the work technically sound?

Yes

Are the conclusions drawn adequately supported by the results?

Partly

Are sufficient details of methods and analysis provided to allow replication by others?

Yes

Reviewer Expertise:

Originally a Biochemist, now Associate Professor of Bioethics with a specific interest in research integrity, ethics and governance.

I confirm that I have read this submission and believe that I have an appropriate level of expertise to confirm that it is of an acceptable scientific standard, however I have significant reservations, as outlined above.

# Reviewer report: Reviewer response for version 1

**DOI:** 10.5256/f1000research.160148.r317744

**Published:** 2024-09-25

**Authors:** Maria Pietilä

**Affiliations:** 1 University of Eastern Finland, Eastern Finland, Finland

Thank you for the invitation to review the paper. The paper addresses an important and timely topic: the potential of narrative CVs in changing the current ways of research assessment. It draws on data gathered at a workshop organized in the Netherlands and data obtained from a pre-workshop survey.

 The paper is well situated in the broader context of research assessment reform. The topic is relevant for readers within the science policy environment in different countries. The paper presents interesting findings which are relevant for the wider scholarly community preparing innovative, responsible ways to assess research/ers.

 I agree with the authors that assessments have for long had narrative elements included. For example, in funding applications researchers have had to put their research proposal in a wider context and to explain its significance to evaluators who may not be experts in the topic. In recruitment, motivation/cover letters represent narratives in which the researchers tell about their skills and career background, often in a free format. Thus, narrative CVs are not completely new in the context of evaluation.

 In the following, I make some comments/reflections/suggestions that the authors may consider when revising their paper and when organizing future workshops on the topic.

 I would consider adding at the beginning of the paper a brief description of narrative CVs: what do they normally look like, what do they aim for, etc.

 The paper places significant (mostly positive) expectations on narrative CVs and maintains an overall optimistic tone toward their use. However, some of the findings in the paper sound more like hypothesis for empirical research. For example, the argument that narrative CVs are good for women and minorities was put forward by the workshop participants. In the paper, you could consider, whether there is a need to critically assess this expectation - is there any empirical research showing that this is the case? I also wonder to what extent narrative CVs in practice lead to the acknowledgement of a broader set of skills. Changing the documentation does not mean that the valuations critical for assessment change. Candidates applying for funding or jobs may still be inclined to present traditional outputs in their narrative CVs (maybe for good reasons). Despite universities/funders’ goal towards identifying and acknowledging a wider set of researchers’ contributions, evaluators may still give priority to traditional outputs and activities at the extent of others. Therefore, the authors might reflect on the need to take into account broader power dynamics in academic recruitment with different actors and priorities and the 'cultural burden' connected to existing research assessment practices that may shape how contributions in narrative CVs are assessed. 

 I would argue organizations using narrative CVs should be encouraged to state clearly how different contributions in narrative CVs are assessed: if all the contributions are of equal value or if some are more valued than others, given the different needs of the funder/employing unit. Objective comparison of candidates’ merits/achievements is crucial for example in recruitment, and thus having transparent criteria and showing them to the applicants is essential (this was touched upon on page 4).

 Therefore, I suggest adding some more reflection on the needs for further academic research on the topic (with proper research designs; such research is already being conducted). Also, the authors are encouraged to consider ‘practice-oriented sessions’ on how to evaluate narrative CVs in the future workshops (incl. how to make comparisons between candidates who bring up different kinds of contributions in their narrative CVs).

Is the work clearly and accurately presented and does it cite the current literature?

Yes

If applicable, is the statistical analysis and its interpretation appropriate?

Not applicable

Are all the source data underlying the results available to ensure full reproducibility?

Yes

Is the study design appropriate and is the work technically sound?

Yes

Are the conclusions drawn adequately supported by the results?

Partly

Are sufficient details of methods and analysis provided to allow replication by others?

Partly

Reviewer Expertise:

Academic careers; research assessment; gender equality in academia; university organisations

I confirm that I have read this submission and believe that I have an appropriate level of expertise to confirm that it is of an acceptable scientific standard, however I have significant reservations, as outlined above.
